# Book Review: The Mind-Body Politic

**DOI:** 10.3389/fpsyg.2021.728578

**Published:** 2021-08-03

**Authors:** Rebecca Zeilstra

**Affiliations:** Department of Philosophy and Religious Studies, Utrecht University, Utrecht, Netherlands

**Keywords:** enactivism, situated cognition, embodied cognition, mind shaping, affective framing, neoliberal capitalism, political philosophy of mind, kantianism

Maiese and Hanna shed light on the political implications of an embodied, enactive, and socially situated philosophy of mind. Their central statement is that because the environment, and in particular institutions, shape our thinking, we should design constructive and enabling social institutions. Neoliberal capitalist institutions do not contribute to human flourishing but are deforming and destructive, according to the authors. Their interdisciplinary approach makes the book worthwhile reading, although it also raises some problems. The authors show that certain aspects of neoliberal capitalist institutions are problematic, but not that we would necessarily be better off without them.

The book's perspective on philosophy of mind entails that both environmental and social structures “*literally shape* our essentially embodied minds” (Maiese and Hanna, 2019, p. 8). The amount of information in our world is overwhelming. To deal with this, animals, and more particularly human animals develop what the authors call affective framing. “Affective framing is a spontaneous, non-inferential, and pre-reflective way of discriminating, filtering, and selecting information that allows us to reduce the overwhelming clutter of information to something first-personally manageable and confer upon it specific cognitive significance” (Maiese and Hanna, 2019, p. 41). For example, the pre-reflective and embodied feeling of fear signals that one should immediately run away from a dangerous predator. The authors suggest that the initial reasons humans developed habitual practices of affective framing are survival as well as well-being and faring well in the social environment. Furthermore, they claim that these practices depend on social processes, and that the individual is partially determined by social processes while she helps to shape them at the same time.

Maiese's and Hanna's Kantian suggestion is that because humans are of intrinsic worth, everyone deserves to be met in their true human needs—basic needs such as eating or social contact—and true humanity realizing needs—more complex needs such as enjoying higher education. The authors claim that if people engage in the right kind of cooperation, they develop habitual practices of affective framing which help satisfying true human and humanity realizing needs. The authors call this “collective wisdom” (Maiese and Hanna, 2019, p. 64). Yet sometimes humans develop habitual practices which seem beneficial on the short term, but which hinder the satisfaction of our needs on the long term. These detrimental habitual practices lead to what the authors call “collective sociopathy” (Maiese and Hanna, 2019, p. 78).

*The Mind-Body Politic* suggests that neoliberal capitalism leads to collective sociopathy because neoliberal capitalist institutions shape human thinking to realize maximum profit instead of satisfying true human and humanity realizing needs. In chapters 5 and 6, case studies on mental health care and university education are used to show that rigid habits—e.g., a rigid egoistic focus on competitiveness—are stimulated through social pressure. The authors argue that the capitalist focus on egoism and self-interest precludes people from engaging in the collaborative aspects of sensemaking. The book's assumption is that if people are not pressured into developing rigid capitalist habits but are allowed to develop flexible habits—e.g., curiosity or imagination—they will rediscover the collaborative practices that lead to healthy forms of sense-making. The authors' concrete suggestions on how to develop flexible habits involve, for instance, empathically mirroring others in play or dancing in pairs.

Although this book touches upon many interesting issues, there are some methodological problems. Firstly, the way the authors prove their claims is not always convincing. They initially claim that they will argue for the destructiveness of capitalist institutions by providing factual evidence, phenomenological observations and thought experiments. However, in many cases, the authors only come up with unargued facts and anecdotal phenomenological observations that are supposed to reveal capitalism's problematic aspects. They do not provide structural proof that modern capitalist institutions have a pervasive detrimental influence on human thinking. For example, the book does not involve extensive socio psychological or neurological studies on how human thinking or behavior develops in societies that shift to the neoliberal capitalist system.

Secondly, Maiese and Hanna tend to ignore that capitalist institutions have their benefits. The book *Factfulness* points out that poverty levels never have been as low as in our current global capitalist system (Rosling et al., 2018). While in 1966, 50 percent of the world population lived in extreme poverty—which means being hungry regularly, sleeping on the floor and having no excess to basic antibiotics—only 9 percent of the population lives in extreme poverty today. It is particularly problematic that Maiese and Hanna do not consider this fact because their anti-capitalist ideas lack insight on economic reform.

Thirdly, it is not clear what is required to change habits, and whether this is a bottom-up or top-down process. The authors' perspective on political philosophy of mind entails that the social environment literally shapes human minds. Therefore, they claim that “we must *change* the social institutions through which habits are instilled in us […] and create *constructive, enabling* social institutions instead” (Maiese and Hanna, 2019, p. 226). The assumption seems to be that habitual patterns of affective framing are shaped top-down, and that people can develop healthy habits only if enabling institutions are in place. However, part of the authors' Kantian perspective is that false habitual believes “need to be *critically and freely taken apart by the rational human agent herself* ” (Maiese and Hanna, 2019, p. 301). People should become aware of their true human and humanity realizing needs, “and then, bottom-up, one designs social institutions whose structure and dynamics are such that they do in fact bring about the satisfaction of those needs” (Maiese and Hanna, 2019, p. 246). The book leaves unclear how people from within destructive institutions can develop insights on how to change their habits, while at the same time these institutions still literally shape their minds.

Nonetheless, *The Mind-Body Politic* is a magnificent book. Albeit the critique that some aspects of capitalism have a detrimental influence on people is well-known, the insight that neoliberal, capitalist institutions have a detrimental influence on our well-being *because* they shape our habitual patterns of thinking and behaving is refreshing. The book should thus not only be read by philosophers, but also by psychologists, economists, and policy makers. For taking a stance in philosophy of mind has political implications, and it is the merit of *The Mind-Body Politic* to make this clear.

## Author Contributions

The author confirms being the sole contributor of this work and has approved it for publication.

## Conflict of Interest

The author declares that the research was conducted in the absence of any commercial or financial relationships that could be construed as a potential conflict of interest.

## Publisher's Note

All claims expressed in this article are solely those of the authors and do not necessarily represent those of their affiliated organizations, or those of the publisher, the editors and the reviewers. Any product that may be evaluated in this article, or claim that may be made by its manufacturer, is not guaranteed or endorsed by the publisher.
